# 17β-Estradiol Abrogates Oxidative Stress and Neuroinflammation after Cortical Stab Wound Injury

**DOI:** 10.3390/antiox10111682

**Published:** 2021-10-25

**Authors:** Kamran Saeed, Myeung Hoon Jo, Jun Sung Park, Sayed Ibrar Alam, Ibrahim Khan, Riaz Ahmad, Amjad Khan, Rahat Ullah, Myeong Ok Kim

**Affiliations:** 1 Division of Life Sciences and Applied Life Science (BK 21 FOUR), College of Natural Science, Gyeongsang National University, Jinju 52828, Korea; kamran.biochem@gnu.ac.kr (K.S.); audgns1217@gnu.ac.kr (M.H.J.); jsp@gnu.ac.kr (J.S.P.); ibrar@gnu.ac.kr (S.I.A.); ibrahim1994@gnu.ac.kr (I.K.); riazk0499@gnu.ac.kr (R.A.); amjadkhan@gnu.ac.kr (A.K.); rahatullah1414@gnu.ac.kr (R.U.); 2 Alz-Dementia Korea Co., Jinju 52828, Korea

**Keywords:** traumatic brain injury, 17β-estradiol, neuroprotection, oxidative stress, neuroinflammation, astrocytosis, microglial polarization, neurodegeneration

## Abstract

Disruptions in brain energy metabolism, oxidative damage, and neuroinflammation are commonly seen in traumatic brain injury (TBI). Microglial activation is the hallmark of neuroinflammation. After brain injury, microglia also act as a double-edged sword with distinctive phenotypic changes. Therefore, therapeutic applications to potentiate microglia towards pro-inflammatory response following brain injury have become the focus of attention in recent years. Here, in the current study, we investigated the hypothesis that 17β-estradiol could rescue the mouse brain against apoptotic cell death and neurodegeneration by suppressing deleterious proinflammatory response probably by abrogating metabolic stress and oxidative damage after brain injury. Male C57BL/6N mice were used to establish a cortical stab wound injury (SWI) model. Immediately after brain injury, the mice were treated with 17β-estradiol (10 mg/kg, once every day via i.p. injection) for one week. Immunoblotting and immunohistochemical analysis was performed to examine the cortical and hippocampal brain regions. For the evaluation of reactive oxygen species (ROS), reduced glutathione (GSH), and oxidized glutathione (GSSG), we used specific kits. Our findings revealed that 17β-estradiol treatment significantly alleviated SWI-induced energy dyshomeostasis and oxidative stress by increasing the activity of phospho-AMPK (Thr172) and by regulating the expression of an antioxidant gene (Nrf2) and cytoprotective enzymes (HO-1 and GSH) to mitigate ROS. Importantly, 17β-estradiol treatment downregulated gliosis and proinflammatory markers (iNOS and CD64) while significantly augmenting an anti-inflammatory response as evidenced by the robust expression of TGF-β and IGF-1 after brain injury. The treatment with 17β-estradiol also reduced inflammatory mediators (Tnf-α, IL-1β, and COX-2) in the injured mouse. Moreover, 17β-estradiol administration rescued p53-associated apoptotic cell death in the SWI model by regulating the expression of Bcl-2 family proteins (Bax and Bcl-2) and caspase-3 activation. Finally, SWI + 17β-estradiol-treated mice illustrated reduced brain lesion volume and enhanced neurotrophic effect and the expression of synaptic proteins. These findings suggest that 17β-estradiol is an effective therapy against the brain secondary injury-induced pathological cascade following trauma, although further studies may be conducted to explore the exact mechanisms.

## 1. Introduction

Traumatic brain injury (TBI) is a universal leading cause of death and permanent disability in children and adults. TBI comprises primary and secondary injury. The primary injury involves direct physical damage to the brain, such as deformation, compression, stretching, shearing, tearing, displacement, and crushing of brain and blood vasculature, causing injury to the vasculature, neural, and glial tissues [1]. Once the primary brain damage occurs, the secondary brain injury further initiates a complicated pathological progression, contributing to the injurious effects in all directions of the initial injury [2,3]. The secondary brain injury promotes oxidative damage, neuroinflammation, and subsequent neurodegeneration [4,5].

There are several pathological hallmarks of TBI including oxidative stress (OS), disruption of the energy homeostasis [6], gliosis, and the suppression of the neurotrophic and synaptic markers [7]. During OS, there is an imbalance between the pro-oxidant and antioxidants, which may result in neural cell death. After the brain injury, a set of oxidative stress markers (lipid peroxides, reactive oxygen) are produced in the brain, while antioxidant enzymes (GSH) are suppressed, which is associated with the pathophysiology of TBI [8]. The enhanced OS causes the suppression of endogenous ROS regulators and their associated genes, such as nuclear factor-erythroid factor 2-related factor 2 (Nrf2), which has been considered as a master transcription factor of antioxidant mechanisms and has become an attractive therapeutic target for the management of various neurological diseases [9]. Regulation of Nrf2 (by using natural or synthetic Nrf2 agonists) has shown pronounced modulatory effects against oxidative stress, neuroinflammation, and neurodegeneration [10,11,12,13,14].

Besides OS, energy imbalance is another contributor to the pathogenesis of neurodegeneration in TBI. One of the known factors p-AMPK (AMP-activated protein kinase) appears to have evolved to maintain cellular energy homeostasis and is activated under low-energy conditions [15]. Prolonged suppression of metabolic activity is commonly associated with secondary brain pathology after TBI [16,17]. AMPK activity is usually reduced after brain injury, contributing to a cellular energy crisis. Therefore, improving AMPK activity may have therapeutic efficacy to cope with secondary brain injury [16]. In complex multicellular organisms, AMPK interacts with hormones and plays a major role in energy intake and expenditure in the body [18].

Brain OS and energy dyshomeostasis have been associated with the activation of microglia and astrocytes, which are key players in initiating an inflammatory response and apoptotic cell death in the CNS after brain injury [19]. During injury, the activated microglia undergo a phenotypic transition, resulting in the activation of numerous microglia phenotypes including disease-associated microglia (DAM). These phenotypes contribute to OS through the persistent generation of ROS and inflammation [13]. The activated microglia after CNS insult produces either pro-inflammatory or anti-inflammatory responses. The pro-inflammatory response involves the production of cytokines, such as tumor necrosis factor α (TNF-α), inducible nitric oxide synthase (iNOS), interleukin-1β (IL-1 β), and interleukin-6, to intensify damage following brain injury, whereas the anti-inflammatory response mediates the neuroprotective effect by the release of transforming growth factor β (TGF-β), insulin growth factor-1 (IGF-1), and interleukin-10 [20,21,22]. The release of inflammatory cytokines and apoptotic cell death may lead to the suppression of the neurotrophins and synaptic markers [23] as several studies have suggested that activation of neurotrophic factors may confer neuroprotection in brain injury [24]. Therefore, switching microglial activation towards an anti-inflammatory response presents a critical strategy to limit brain damage following TBI [25,26].

Previously, several natural and synthetic compounds have been used against the multi-faceted pathological features of brain injury-induced neurodegeneration [27]. It has been well established that estrogen has critical effects on reproductive behaviors, but estradiol, which is the most active estrogen, has prominent effects against neurodegenerative diseases [28,29]. Estradiol effectively increases cognition, memory formation, motor coordination, pain sensitivity, and neurodevelopment [30,31]. Moreover, 17β-estradiol i.p. administration for seven consecutive days increased axonal regeneration after brain injury [32]. Recently, we reported that 17β-estradiol protects neonatal rats’ brains against glutamate-induced neurodegeneration by exerting antioxidant and anti-inflammatory effects [33]. Here, we conducted this study to elucidate the effects of 17β-estradiol against cortical stab wound injury (SWI) injury-induced energy dyshomeostasis, oxidative damage, neuroinflammation, and neurodegeneration.

## 2. Materials and Methods

### 2.1. Animals

Male C57BL/6N mice (10 weeks, average body weight of 25–30 g) were purchased from Samtako Bio, Osan, Korea. The animals were retained randomly in a group of four/cage and carefully acclimatized for one week under a 12 h light/dark cycle at 23–25 °C with 60 ± 10% humidity and free access to food and water at Gyeongsang National University, South Korea. All the experiments involving animals were implemented in agreement with guidelines and principles approved by the Institutional Animal Care and Use Committee (IACUC), Division of Applied Life Sciences, Gyeongsang National University, South Korea (Approval ID: 125).

### 2.2. Animal Model and Drug Administration

The stab wound cortical brain injury (SWI) mouse model was established as previously described with minor changes [34]. In brief, all the mice were anaesthetized with 0.05 mL and 0.1 mL per 100 g of body weight of Zoletil and Rompun, respectively. The mouse was kept on a stereotaxic frame and the skull was exposed by creating a mid-longitudinal incision. A dental drill was used to make a circular craniotomy (4 mm in diameter, 2 mm lateral to the midline, and 1 mm posterior to the bregma) in the mouse skull. Next, to create the stab wound injury, a sharp edge scalpel blade was used and inserted (3 mm, right hemisphere) for 1 min and then removed carefully. Bone wax was used to cover the rupture wound area and the skull was closed carefully with silk suture. The control group (animals) was anaesthetized and surgically prepared but did not receive craniotomy. The mice were subjected to warm temperature by providing continuous heating with a heating lamp until the mice were fully recovered from anesthesia.

Mice were randomly divided into three different groups (*n* = 8 mice/group): control group (naive C57BL/6J mice), SWI group (mice that received injury + saline as a vehicle), and SWI + 17β-estradiol group (injured mice administered with a daily i.p. injection of 10 mg/kg of 17β-estradiol (Sigma Aldrich, Madison, WI, USA) in normal saline for 1 week). Mice were sacrificed after the last i.p. injection for biochemical and histological examination.

### 2.3. Protein Extraction for Biochemical Analysis

For biochemical analysis, the mice were anaesthetized, and the brain was removed immediately. The brain tissue (cortex and hippocampus) was carefully separated and stored at –80 °C. For Western blot analysis, the brain tissues were lysed in PRO-PREP^TM^ protein extraction solution according to the instructions of the manufacturer (iNtRON Biotechnology, Gyeonggi-do, 13202, Korea). Proteins were extracted from the lysed solutions by centrifugation at a speed of 13,000× *g* rpm at 4 °C for 25 min. The supernatants were collected and stored at –80 °C for further analysis. For the separation of nuclear and cytoplasmic fractions, the nuclear and cytoplasmic protein extraction kit (catalog #K266, Biovision Incorporated, Milpitas Boulevard, Milpitas, CA, USA) was used according to the manufacturer’s guidelines.

### 2.4. Immunoblotting

For immunoblot analysis, the proteins were quantified using a Bradford assay (catalog #5000006, Bio-Rad Protein Assay kit, Bio-Rad Laboratories, Hercules, CA, USA) as described previously [35]. An equal volume of 20 µg of proteins was mixed with a 2× Sample Buffer (Invitrogen, Carlsbad, CA, USA). Proteins were separated using 10% SDS-PAGE and then transferred onto polyvinylidene difluoride membranes (PVDF) (Immobilon-PSQ, Merck Millipore, Burlington, MA, USA). After transfer, the membranes were blocked with 5% skimmed milk and incubated with the primary antibodies at 4 °C overnight. The next day, the PVDF membranes were incubated for 1 h with respective secondary antibodies. The protein bands were detected using enhanced chemiluminescence (ECL) reagent (EzWestLumiOne, ATTO, Tokyo, Japan). The bands were attained using X-ray films without exposure to external light. The bands were quantified via ImageJ software and the data were analyzed and graphs were created using GraphPad Prism software (ver. 8.0.2, San Diego, CA 92108, USA).

### 2.5. Brain Tissue Preparation and Sectioning

For immunohistochemistry (IHC) and morphological analysis, the experimental mice were anaesthetized and transcardially perfused with 0.9% normal saline solution and 4% paraformaldehyde, respectively. The whole brain was removed carefully and fixed at 4 °C in ice-cold paraformaldehyde for 72 h. The brain was placed in 20% sucrose PBS solution for 48 h. Next, the brain was fixed by freezing vertically in optimum cutting temperature (O.C.T) compound (tissue-Tek O.C.T compound medium, Sakura Finetek USA, Inc., Torrance, CA, USA). Then, 14-µm brain coronal sections were taken with a CM3050C cryostat microtome (Leica, Nussloch, Germany) and collected on polarized slides and mounted.

### 2.6. Immunofluorescence Analysis

The immunofluorescence staining was performed as described previously with slight modification [36]. In brief, the brain slices were dried at room temperature before staining, rinsed in phosphate-buffered saline (PBS), treated with proteinase-K, and blocked for 60 min in 5% normal goat serum followed by incubation with primary antibodies (dilution, 1:100) overnight at 4 °C. The slides were then washed with 1% PBS and incubated for a maximum of 2 h at room temperature with secondary fluorescein isothiocyanate (FITC)- or tetramethylrhodamine isothiocyanate (TRITC)-conjugated antibodies. The tissue slides were washed again and 4′,6- diamidino-2-phenylindole (DAPI) was poured for nucleus detection for 8–10 min. A fluorescent mounting medium (FluoView FV 1000; Olympus, Tokyo, Japan) was used for mounting the slides and images were taken by a confocal scanning microscope (FV1000MPE). The fluorescent intensity of the images was quantified with ImageJ and the data were analyzed with GraphPad Prism software. Iba-1- and caspase-3-positive cell bodies stained with DAPI were counted manually and reviewed morphologically.

### 2.7. Immunohistochemistry

The IHC was performed as we have described previously [37]. Briefly, the brain slides were washed twice with PBS for 5 min followed by incubation with proteinase K (20 mg/mL) for 10 min at room temperature. The slides were quenched in a 9:1 solution of methanol and hydrogen peroxide for 10 min. This was followed by blocking the slides with normal goat serum containing 5% BSA and 0.3% triton X-100 for 1.5 h. The slides were incubated overnight at 4 °C with primary anti-Iba-1 antibody followed by incubation with goat anti-rabbit biotinylated secondary antibody for 1 h and subsequently treated with ABC reagents (Standard VECTASTAIN ABC Elite Kit; Vector Laboratories, Burlingame, CA) for 1 h at room temperature in the dark. The brain slices were washed with PBS twice for 5 min and incubated in 3,3′-diaminobenzidine tetrahydrochloride (DAB) for 1 min. The sections were washed with distilled water and dehydrated in 50, 70, and 95% ethanol solution, respectively, and cleared in xylene solution for 5 min and mounted. The brain morphology of the stained slides was analyzed using a microscope (Zeiss Axioskop 2 Plus).

### 2.8. Lesion Volume Assessment

Nissl staining was used to analyze the lesion volume of injured mice brains. Briefly, the brain sections were washed with PBS and stained for 20 min using 0.1% cresyl violet solution. Stained slices were rinsed in distilled water and differentiated in 70, 95, and 100% ethanol, respectively, for 15 min. The slides were then cleared twice in 100% xylene and mounted for cortical lesion analysis as previously reported [38].

### 2.9. Antibodies

The primary antibodies used in Western blot, immunofluorescence, and IHC are presented in Table 1. For secondary antibodies, either goat anti-rabbit or goat anti-mouse horseradish peroxidase were used (dilution 1:10,000), purchased from Cell Signaling Technology (Danvers, MA, USA) and Santa Cruz Biotech (Dallas, TX, USA), respectively.

### 2.10. Reactive Oxygen Species (ROS) and Glutathione Assay

To measure ROS activity, a fluorometric assay involving 2′,7′-Dichlorodihydrofluorescein diacetate (H2DCFDA) was used as previously described [39]. To assess the total level of GSH and quantified GSH/GSSG enzyme levels, a glutathione fluorometric assay kit (BioVision, Milpitas Boulevard, Milpitas, CA, USA, Catalog #: K264-100) was used according to the manufacturer’s instructions.

### 2.11. Glutathione S-Transferase (GST) and Glutathione Reductase (GR) Assay

To measure GST and GR activities in cortical and hippocampal brain tissue, the GST Colorimetric Activity Assay Kit (Bio vision, Milpitas Boulevard, Milpitas, CA, USA, Catalog #: K263) and Glutathione Reductase Activity Colorimetric Assay Kit (Bio Vision, Milpitas Boulevard, Milpitas, CA, USA, Catalog #: K761) was used according to the manufacturer’s guidelines.

### 2.12. Statistical Analysis

Western blot band densities (scanned X-ray films) were measured via ImageJ software (1.51w, NIH, Bethesda, MD, USA). Immunofluorescence Tiff files were analyzed by integrated densities using ImageJ software or by manually counting the number of DAPI-stained positive cells. GraphPad Prism (ver.8.0.2, San Diego, CA, USA) was used to evaluate data. Statistical significance was measured by using a one-way analysis of variance (ANOVA), followed by Tukey’s post-hoc analysis. The statistical values were calculated as the mean ±S.E.M. A *p*-value ≤ 0.05 was considered statistically significant. The significance difference presented as * *p* ≤ 0.05 for the control vs. SWI group and ^#^ *p* ≤ 0.05 for the SWI vs. SWI + 17β-estradiol group. Statistical analysis is conferred in the figure legends.

## 3. Results

### 3.1. 17β-Estradiol Alleviates Energy Dyshomeostasis and Oxidative Stress after Brain Injury

**Figure 1:**
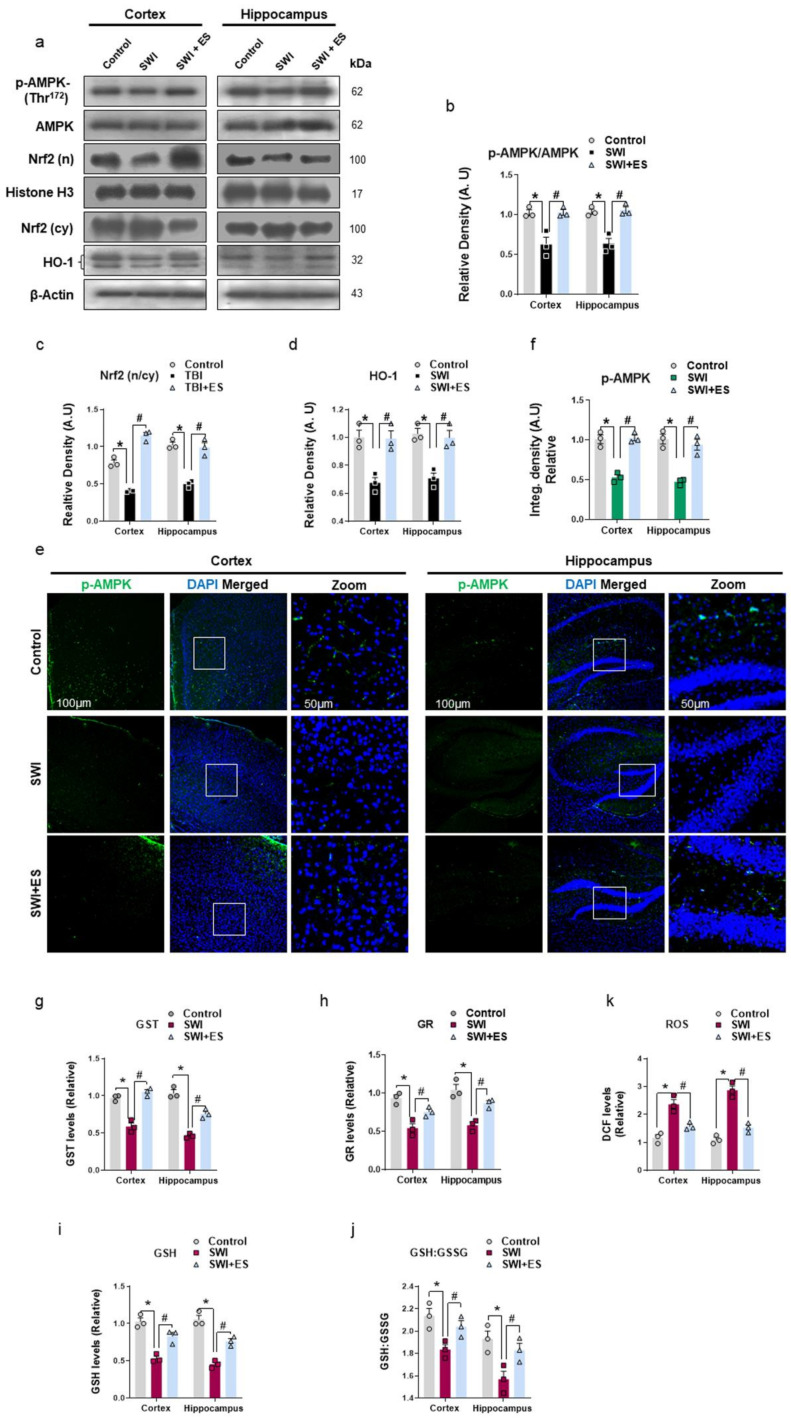
17-β estradiol (ES) alleviates metabolic and oxidative stress after SWI. (**a**–**d**) Western blot analysis showing protein expression of P-AMPK/AMPK, Nrf2 (nuclear)/Nrf2 (cytoplasmic), and HO-1 in the cortex and hippocampal region. Anti-β-actin/anti-Histone H3 was used as a control for protein levels across the samples. (**e**,**f**) Brain sections from the experimental animals revealing P-AMPK (green) immunoreactivity stained with DAPI (blue) within the cortex and DG region, with the respective histogram. The densities are expressed in arbitrary units (AU). Values are presented as means ± S.E.M. (**g**–**k**) Quantitative analysis of GST, GR, GSH, GSH: GSSG, and ROS assay respectively of cortical and hippocampal brain tissue lysate in the experimental groups. The data presented are relative to the control group. *p*-value < 0.05 was considered significant. * p < 0.05 vs. control group, *^#^ p <* 0.05 vs. SWI + 17-β estradiol group. Bar = 50 μm. Statistical significance was determined by one-way analysis of variance (ANOVA) followed by Tukey’s post-hoc analysis using the GraphPad Prism (ver. 8.0.2) software.

Disruptions in brain energy metabolism and oxidative damage are commonly seen in traumatic brain injury (TBI) [6,8]. After brain injury, there is a switch from aerobic to anaerobic metabolism, which is less energy efficient and leads to the accumulation of free radicals [40]. Therefore, to examine the phosphorylation status of AMPK (Thr172) associated with cellular energy hemostasis after brain injury, we carried out Western blot and immunofluorescence analysis of the cortical and hippocampal regions. The results revealed the downregulation of AMPK phosphorylation (normalized to total-AMPK) after brain injury compared to the control group (Figure 1a,b). The immunofluorescence analysis of injured brain slices further substantiated the loss of p-AMPK immunoreactivity within the cortex and hippocampal DG region (Figure 1e,f). However, post-injury treatment of 17β-estradiol significantly increased the expression and immunoreactivity of p-AMPK compared to the control group (Figure 1a,b,e,f). Moreover, the cortical and hippocampal tissue lysate of injured mice revealed alteration in cellular redox hemostasis as illustrated by a prominent loss in antioxidant protein expression of nuclear Nrf2 and HO-1 (Figure 1a,c,d). This was accompanied by the depleted levels of glutathione S-transferase (GST), glutathione reductase (GR), glutathione (GSH) enzymes, and quantified GSH/GSSG values and the accumulation of reactive oxygen species (ROS) in the SWI model (Figure 1g–i). On the contrary, estradiol treatment upregulated the expression of Nrf2 and HO-1 (Figure 1a,c,d) and enhanced the GST, GR, GSH and GSH/GSSH cellular stores and reduced the ROS production as revealed by low DCF intensities when compared to the control group (Figure 1g–i). These data demonstrate that post-injury administration of 17β-estradiol may alleviate energy crisis and cellular redox dyshomeostasis observed in traumatized mouse brains.

### 3.2. 17β-Estradiol Inhibits Gliosis and Inflammatory Response after Brain Injury

**Figure 2:**
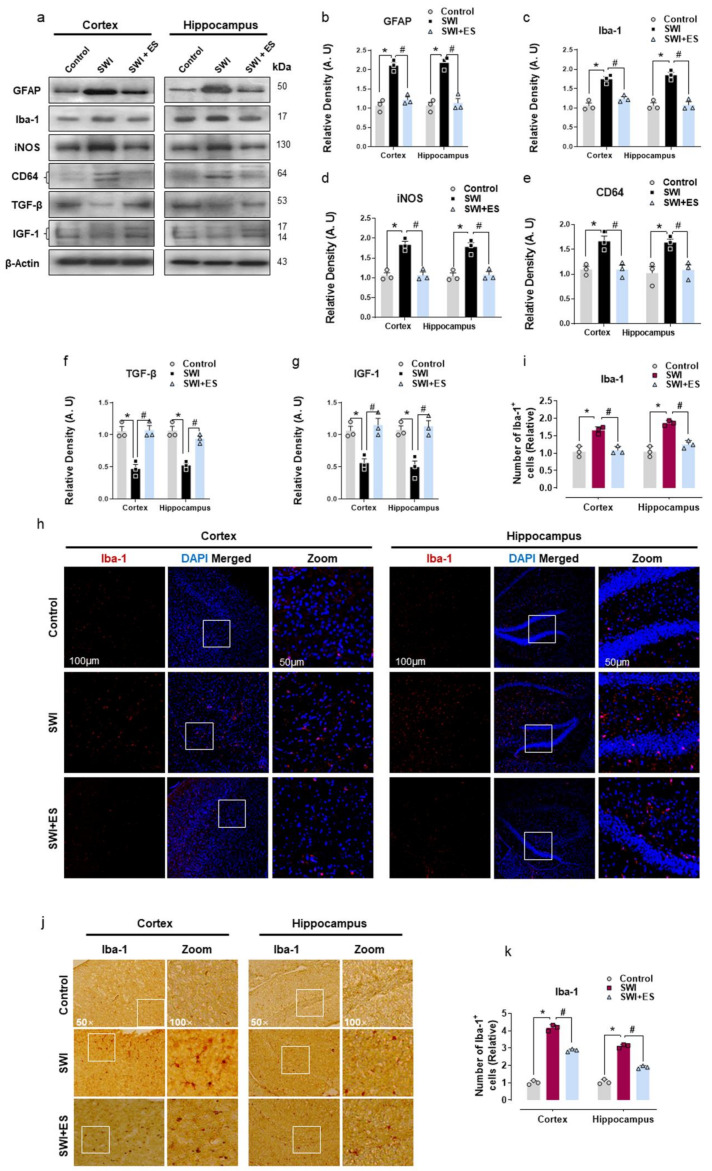
17-β estradiol (ES) impedes gliosis and brain inflammation after SWI. (**a**–**g**) Western blot analysis showing protein expression of GFAP, Iba-1, CD64, TGF-β, and IGF-1 in the experimental groups. Anti-β-actin was used as a control for the protein levels across the samples. The densities are expressed in arbitrary units (AU). (**h**,**i**) Brain section from the experimental animals illustrating Iba-1-positive cells (red) stained with DAPI (blue) of the cortex and DG region, with the respective histogram. (**j**,**k**) Iba-1 immunostained sections of the cortex and hippocampal-DG region of the experimental groups with respective histograms. Values are presented as means ± S.E.M. The data presented are relative to the control group. *p*-value < 0.05 was considered significant. * p < 0.05 vs. control group, *^#^ p <* 0.05 vs. SWI + 17-β estradiol group. Bar = 50 μm. Statistical significance was determined by one-way analysis of variance (ANOVA) followed by Tukey’s post-hoc analysis using the GraphPad Prism (ver. 8.0.2) software.

Astrocyte and microglial cells of the CNS are key players in mediating neuroinflammatory response after brain injury [20]. Many studies have demonstrated that a shift in metabolic state and oxidative stress is often associated with glial cell activation [6,13,41]. Therefore, we further investigated astrogliosis and microglial activation. Our Western blot analysis revealed increased expression of GFAP and Iba-1 protein in the cortical and hippocampal tissue of injured mice (Figure 2a–c). Moreover, the results also showed that the expression of pro-inflammatory markers (CD64 and iNOS) was significantly increased, whereas anti-inflammatory markers (TGF-β and IGF-1) were downregulated after brain injury when compared to the control mouse cohort (Figure 2a,d–g). The immunofluorescence and immunohistochemistry analysis of the brain slices also displayed an increased number of Iba-1-positive cells (Iba-1^+^) within the cortex and hippocampal-DG region of injured mice (Figure 2h–k). However, treatment with 17β-estradiol significantly attenuated gliosis as indicated by reduced GFAP expression and Iba-1 expression and immunoreactivity in the cortical and hippocampal region after brain injury (Figure 2a–c,h–k). Importantly, 17β-estradiol administration downregulated the expression of CD64 and iNOS and increased the expression of TGF-β and IGF-1 (Figure 2a,d–g). These data establish that 17β-estradiol could protect the brain from secondary injury by promoting glial cell polarization from the pro-inflammatory into the anti-inflammatory type.

### 3.3. 17β-Estradiol Restrains Inflammatory Factors in the Injured Mouse Brain

**Figure 3:**
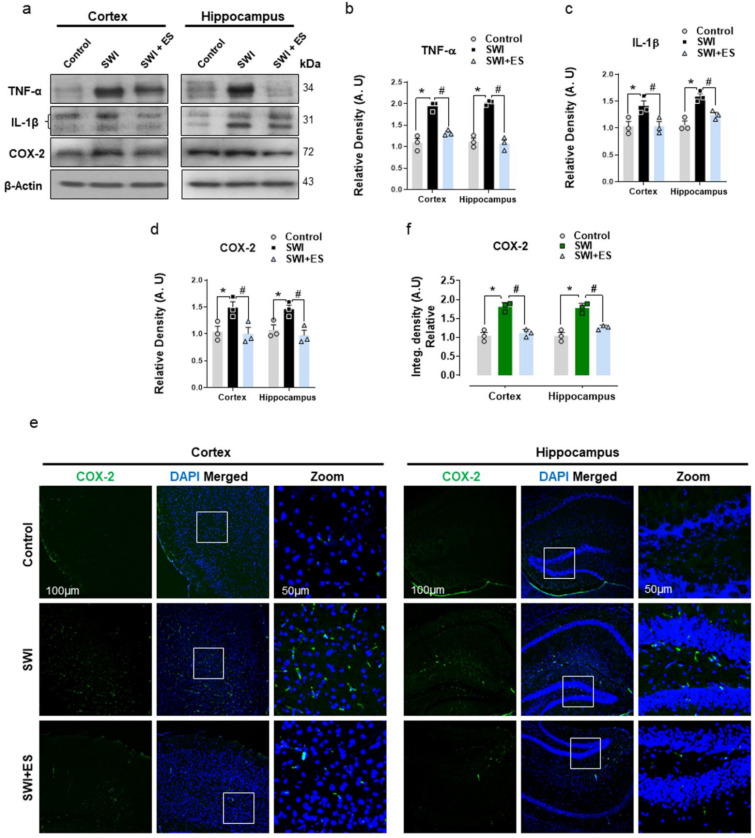
17-β estradiol (ES) inhibits neuroinflammatory mediators after SWI. (**a**–**d**) Western blot analysis showing protein expression of Tnf-α, IL-1β, and COX-2 in the experimental groups. Anti-β-actin was used as a control for protein levels across the samples. (**e**,**f**) Representative of COX-2 (green) immunoreactivity stained with DAPI (blue) in the cortex and DG region, with the respective histogram. The densities are expressed in arbitrary units (AU). Values are presented as means ± S.E.M. The data presented are relative to the control group. *p*-value < 0.05 was considered significant. * p < 0.05 vs. control group, *^#^ p <* 0.05 vs. SWI + 17-β estradiol group. Bar = 50 μm. Statistical significance was determined by one-way analysis of variance (ANOVA) followed by Tukey’s post-hoc analysis using the GraphPad Prism (ver. 8.0.2) software.

Glial cells modulate the neuroinflammatory response in the CNS by releasing pro-inflammatory cytokines to intensify the damage sustained following TBI [42]. Therefore, we further investigated the expression of inflammatory mediators in the cortical and hippocampal brain regions. Western blot analysis revealed prominent expression of TNF-α, IL-1β, and COX-2 in the SWI group (Figure 3a–d). The confocal microscopy also revealed increased COX-2 immunoreactivity within the cortex and hippocampal-DG region after brain injury (Figure 3e,f). Treatment with 17β-estradiol restrained neuroinflammation by reducing the expression of TNF-α, IL-1β, and COX-2 in the injured group when compared to the control cohort (Figure 3a–f). These data illuminate that 17β-estradiol treatment could alleviate glial cell-induced activation of the pro-inflammatory response after brain injury.

### 3.4. 17β-Estradiol Rescued Neuronal Apoptosis and Cell Death after Brain Injury

**Figure 4:**
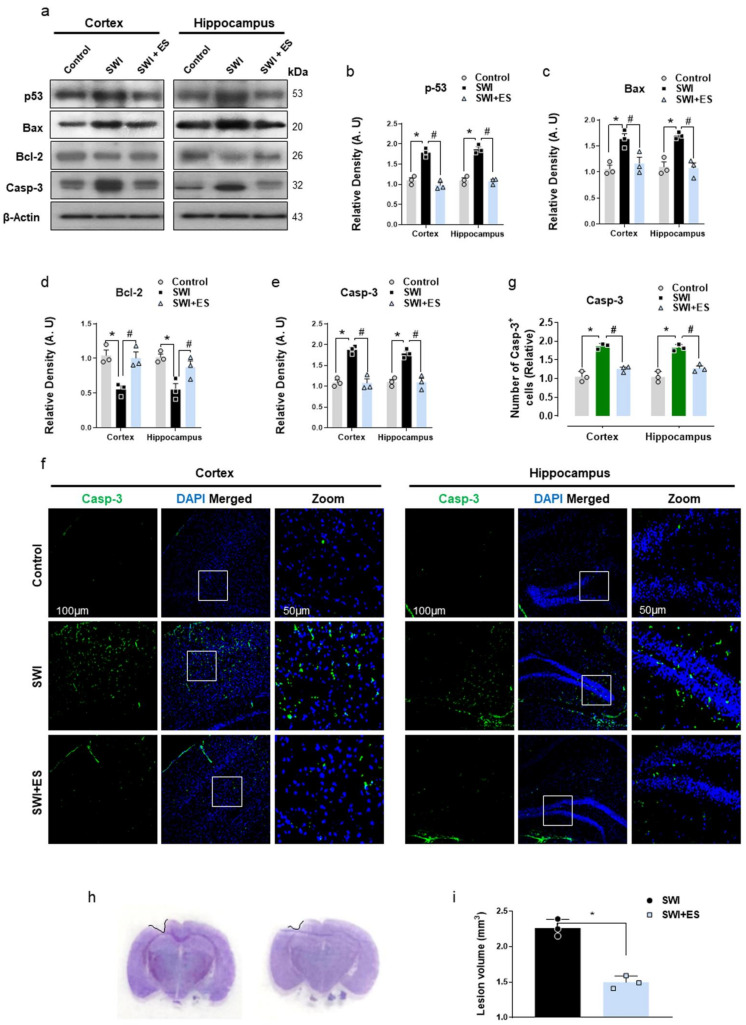
17-β estradiol (ES) reduces cell death and neuronal apoptosis after SWI. (**a**–**e**) Western blot analysis showing protein expression of p-53, Bax, Bcl-2, and caspase-3 in the experimental groups. Anti-β-actin was used as a control for protein levels across the samples. The densities are expressed in arbitrary units (AU). (**f**,**g**) Brain slices from experimental animals revealing caspase-3 (green)-positive cells immunoreactivity stained with DAPI (blue) in the cortex and DG region. (**h**,**i**) Quantification of brain lesion volume at 7 days post-trauma in the SWI + ES-treated mice compared to the SWI group. Values are presented as means ± S.E.M. The data presented are relative to the control group. *p*-value < 0.05 was considered significant. * p < 0.05 vs. control group, *^#^ p <* 0.05 vs. SWI + 17-β estradiol group. Bar = 50 μm. Statistical significance was determined by one-way analysis of variance (ANOVA) followed by Tukey’s post-hoc analysis using the GraphPad Prism (ver. 8.0.2) software.

Glial cell hyperactivation-mediated neuroinflammation plays an important role in TBI-induced neuronal apoptosis and cell death [43]. Therefore, we evaluated the protein expression of apoptosis mediators after brain injury. As revealed by immunoblot, the injured mice displayed activation of p53-mediated neuronal apoptosis and cell death as indicated by increased expression of pro-apoptotic (Bax) and downregulation of anti-apoptotic (Bcl-2) Bcl-2 family proteins when compared to the control mouse cohort (Figure 4a–d). The dysregulation in Bcl-2 family members was associated with the activation of caspase-3 in the SWI group (Figure 4a,e). However, treatment of injured mice with 17β-estradiol significantly mitigated the activation of apoptotic machinery by regulating the expression of p53, Bax, Bcl-2, and caspase-3 in the cortical and hippocampal brain regions (Figure 4a–e). Additionally, the immunofluorescence analysis also revealed enhanced caspase-3 immunoreactivity in the SWI group (Figure 4f,g). On the contrary, injured mice that received 17β-estradiol treatment displayed a reduced number of caspase-3^+^ cells in the cortical and hippocampal regions (Figure 4f,g). Furthermore, the cresyl violet-stained brain sections also revealed a reduced lesion volume in the SWI + 17β-estradiol-treated group compared to SWI on day 7 (Figure 4h,i). These results indicate that 17β-estradiol could rescue TBI-induced neuronal apoptosis and cell death.

### 3.5. 17β-Estradiol Enhanced the Neurotrophic Effect and the Expression of Synaptic Proteins after Brain Injury

**Figure 5:**
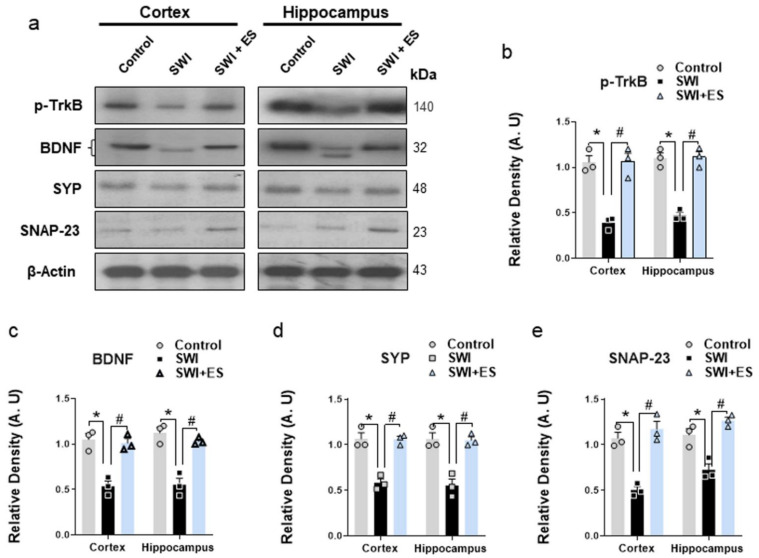
17-β estradiol improves neurotrophic and synaptic functions after SWI. (**a–e**) Western blot analysis showing the protein expression of p-TrkB, BDNF, SNAP-23, and SYP in the experimental groups. Anti-β-actin was used as a control for protein levels across the samples. The densities are expressed in arbitrary units (AU). Values are presented as means ± S.E.M. The data presented are relative to the control group. *p*-value < 0.05 was considered significant. * p < 0.05 vs. control group, *^#^ p* < 0.05 vs. SWI + 17-β estradiol group. Bar = 50 μm. Statistical significance was determined by one-way analysis of variance (ANOVA) followed by Tukey’s post-hoc analysis using the GraphPad Prism (ver. 8.0.2) software.

Neurotrophic factors play an important role in synaptic function, neuronal growth, and survival to protect and recover brain functions following TBI [44,45]. Our results revealed disruptions of the neurotrophic effects after brain injury as illustrated by the loss of neurotrophin BDNF and its receptor phospho-TrkB expression (Figure 5a–c). The injured mice also showed a loss of synaptic density proteins (SNAP-23 and SYP) when compared to the control group (Figure 5a,d,e). However, the 17β-estradiol + SWI group revealed an improved neurotrophic effect and synaptic function as illustrated by the increased expression of BDNF, p-TrkB, and SNAP-23 and SYP in the cortical and hippocampal brain regions compared to injured mice (Figure 5a–e). The data demonstrate that 17β-estradiol administration could improve the recovery of neuronal functions and synaptic protein levels after brain injury.

## 4. Discussion

Herein, we showed that 17β-estradiol administration after cortical stab wound injury (SWI) significantly alleviated neuronal loss and cell death by improving energy hemostasis, abrogating oxidative stress (OS) and neuroinflammation. Importantly, 17β-estradiol reduced the gliosis and proinflammatory response after brain injury as illustrated by the higher expression of iNOS, CD64, Tnf-α, and COX-2 and lower expression of TGF-β and IGF-1, respectively. In addition, the treatment also enhanced the neurotrophic effect and synaptic protein density in the experimental mice’s brains after injury.

Multifaceted pathological features of TBI include energy crisis, oxidative stress, neuroinflammation, neuronal apoptosis, and synaptic dysfunctions [7], and regulation of these effects in TBI has shown rescuing effects against brain injury-induced neurodegeneration [46]. To uncover the effects of 17β-estradiol against the energy imbalance and oxidative stress (OS), we analyzed the expressions of p-AMPK, antioxidant proteins and cytoprotective enzymes, and ROS levels. According to our findings, there was a significant downregulation of the p-AMPK/AMPK ratio after brain injury in the cortical and hippocampal brain region as monitored by phosphorylation on Thr172 by immunoblot analysis and confocal microscopy. AMPK is a conserved signaling kinase and indicator of cellular energy status in the body [47]. The kinase is activated via phosphorylation when ATP levels are depleted, which makes the p-AMPK/AMPK ratio a good marker of neuronal responsiveness to alteration in the cellular energy status [48,49]. It has been reported that the activity of AMPK decreases after controlled cortical impact (CCI) in the cortex and ipsilateral hippocampus by 24 h post-injury [16]. The role of 17β-estradiol in maintaining energy hemostasis after SWI is apparent in our data as illustrated by a significant increase in the p-AMPK/AMPK ratio. Furthermore, brain injury is associated with depressed aerobic metabolism, which leads to the accumulation of free radicals [40]. Our SWI mouse model revealed an accumulation of brain ROS, depleted stores of glutathione S-transferase (GST), glutathione reductase (GR), reduced glutathione (GSH), and low values of quantified GSH/GSSG levels. The expression of Nrf2 and its ARE-mediated downstream HO-1 protein expression was also downregulated, indicating an increased brain OS. The suppression of p-AMPK and Nrf2 in TBI-induced mice is in accordance with the previous studies conducted by our group [7]. Interestingly, the expression of Nrf2 and HO-1 was significantly upregulated in SWI + 17β-estradiol-treated brains, and this was also accompanied by the significantly low levels of ROS, and high activities of GST, GR, and GSH and enhanced GSH/GSSG values. Estradiol has been previously reported to regulate AMPK activity to protect neurons against ischemic stroke [50] as well as suppress oxidative stress in both male and female rats [51]. Nrf2 shows great promise as a molecular target in TBI. Nrf2/ARE signaling plays an important role in the maintenance of the redox state for the defense of intracellular OS [52]. Nrf2 (nuclear), when combined with the ARE, activates a batch of endogenous substances, such as heme oxygenase-1 (HO-1), NAD(P)H: quinine oxidoreductase-1 (NQO1), peroxidase (GPx), GST, and GR, to regulate cellular redox balance [53,54,55]. Many studies have reported the protective role of Nrf2/ARE signaling pathway activation after brain injury [56,57,58,59]. Several studies indicate crosstalk between Nrf2 and estrogen [60,61,62]. Previously, it has been reported that activation of Nrf2 by 17β-estradiol alleviates brain injury, as when Nrf2 was knocked out, 17β-estradiol lost its anti-inflammatory effects [63]. These findings suggest the probable activation of the Nrf2/ARE pathway by17β-estradiol to regulate energy dyshomeostasis and OS after brain injury.

The OS and alteration in energy homeostasis contribute to the activation of astrocytes and microglia [64,65]. Several reports have revealed that the neuroinflammatory response is key to neuronal damage in the acute stage of TBI [66]. Both astrocytes and microglia after brain injury may be activated immediately, causing neuroinflammation and brain damage [67]. The activated microglia after brain injury and in other neurological disorders act as a double-edged sword by forming a continuum state with distinct phenotypes, involved in the process of neurodegeneration and repair [68,69,70]. Importantly, the current study revealed enhanced GFAP and Iba-1 expression after brain injury. Moreover, following SWI, glial cells revealed increased polarization towards the proinflammatory state as identified by the upregulated expression of iNOS [71] and CD64 [72], and downregulated expression of anti-inflammatory markers (TGF-β and IGF-1) [73,74]. 17β-estradiol administration prominently reduced the glial cell-mediated inflammatory response. Estrogens play an important role in controlling microglia activity [75]. Sex hormone has previously been reported by others to reduce brain inflammation after ischemic stroke [76,77]. In line with our study, Jin Wang et al. reported that 17β-estradiol inhibits microglia-mediated A1 astrocytic activation to alleviate neuroinflammation in a mouse TBI model [78]. Indeed, numerous studies have recently reported that potentiating the immune response of glial cells from a pro-inflammatory into an anti-inflammatory phenotype alleviates pathological discrepancies in many neurodegenerative disorders [67,68,79,80,81]. Collectively, these findings indicate that 17β-estradiol may exert a neuroprotective effect after brain injury through modulation of the glial cell-mediated inflammatory response.

Glial cell activation can upregulate various inflammatory factors and cytokines, resulting in dysfunction of the CNS [82,83,84]. Microglia isolated from the TBI brain (ex vivo) have been shown to initiate neuroinflammation after being stereotactically injected into the cortex of naive animals [85]. The inflammatory response mediates the upregulation of pro-inflammatory mediators like Tnf-α, IL-1β, and inducible iNOS, which may contribute to neuronal injury following TBI [86,87,88]. Moreover, cyclooxygenase-2 (COX-2) induction has been associated with post-traumatic neuroinflammation and cell death [89,90]. Our result showed enhanced expression of TNF-α and IL-1β and COX-2 in the SWI model. The upregulation of TNF-α expression has been associated with the activation of microglia [91]. In the current study, we demonstrated that 17β-estradiol lowered the expression of these markers and attenuated the inflammatory response in the cortical and hippocampal brain region after injury. Sex-steroid hormones have previously been known to dampen brain-intrinsic immune responses in different neurological disorders [92,93,94]. Many studies have reported that 17β-estradiol can prevent the production and secretion of pro-inflammatory cytokines, such as TNFα, IL-1β, and IL-6 [95]. Recently, we reported that 17β-estradiol alleviates neuroinflammation in developing rats’ brains after glutamate-induced excitotoxicity [33].

The increased expression of the inflammatory cytokines is a trigger for mitochondrial dyshomeostasis and apoptotic cell death [19]. Post-traumatic neurodegeneration correlates with enhanced expression of p53 tumor-suppressor protein [96,97]. p53 mediates apoptosis directly by regulating Bcl-2 family proteins (Puma, Bax, and Bcl-2), inducing mitochondrial membrane permeabilization, releasing cytochrome C, and finally triggering caspase-3 activation and apoptosis [98,99,100,101]. The current data illustrates similar and possible evidence of the p53-mediated neuronal apoptosis associated with deregulated Bcl-2 family protein and caspase-3 activation after brain injury. Interestingly, the administration of 17β-estradiol reduced the expression of these markers and mitigated neurodegeneration. Further, 17β-estradiol has been reported to alleviate neuronal loss after brain injury [32]. In accordance with current studies, recent reports revealed that estradiol protects mitochondrial integrity and prevents the leakage of cytochrome c from mitochondria, therefore preventing the onset of apoptosis [102,103]. These findings indicate that 17β-estradiol might play an important role in reducing cell death and neuronal apoptosis after brain trauma.

Finally, we analyzed the expression of neurotrophic factors and synaptic proteins in the experimental mice brains. Neurotrophin, such as brain-derived neurotrophic factor (BDNF), is actively produced in the brain and is involved in regulating neuronal activity, protection, and day-to-day physiological functions [44]. During injury, it has been suggested that the normal levels of neurotrophic factors [45,104] and synapse proteins are dysregulated [105,106]. Our findings revealed reduced expression of neurotrophic factor (BDNF) and its receptor (TrkB) and downregulated synaptic protein (SNAP-23 and SYP) expression in the SWI model. However, the SWI + 17β-estradiol group illustrated an improved neurotrophic effect and synaptic protein density. In accordance with this, many studies have reported the regulating effects of 17β-estradiol on neurotrophic factors [107,108,109]. These collective findings suggested that 17β-estradiol has strong therapeutic efficacy against the detrimental effects following brain injury.

## 5. Conclusions

In summary, these findings demonstrate that 17β-estradiol treatment suppresses energy crisis, abrogates oxidative stress, and attenuates neuroinflammation after cortical stab wound injury. Furthermore, 17β-estradiol enhanced neurotrophic effects and improved neuronal survival and synapses after brain injury. The neuroprotective and anti-inflammatory effects of 17β-estradiol after brain injury may be attributed to the activation of the Nrf2-ARE pathway. Future mechanistic studies are warranted to elucidate the exact mechanism involved in mediating the neuroprotective effect of 17β-estradiol.

## Figures and Tables

**Table 1:** List of primary antibodies used in immunoblotting and immunofluorescence analysis.

Antibody	Host	Product Identifier	Application (Conc.)	Manufacturer
p-AMPK	Rabbit	#2535S	WB (1:1000), IF (1:100)	Cell Signaling
AMPK	=	#2603S	WB (1:1000)	=
Nrf2	Mouse	SC-722	WB (1:1000)	Santa Cruz
Histon H3	Rabbit	ab1791	WB (1:1000)	Abcam
HO-1	Mouse	SC-136961	WB (1:1000)	Santa Cruz
GFAP	=	SC-33673	WB (1:1000)	=
Iba-1	=	SC-32725	WB (1:1000)	=
Iba-1	Rabbit	PA5-27436	IF (1:100), IHC (1:100)	Thermo Fisher
iNOS	Mouse	SC-7271	WB (1:1000)	Santa Cruz
CD64	=	SC-515431	WB (1:10,000)	=
TGF-β	Rabbit	MA5-15065	WB (1:1000)	Thermo Fisher
IGF-1	Rabbit	ab133542	WB (1:1000)	Abcam
IL1-β	Mouse	SC-32294	WB (1:1000)	Santa Cruz
TNF-α	=	SC-52746	WB (1:1000)	=
COX-2	=	SC-376861	WB (1:1000), IF (1:100)	=
p-TrkB	=	SC-8058	WB (1:1000)	=
BDNF	=	SC-65514	WB (1:1000)	=
SNAP-23	=	SC-374215	WB (1:1000)	=
SYP	=	SC 17750	WB (1:1000)	=
p-53	=	SC-126	WB (1:1000)	=
Bax	Rabbit	2772S	WB (1:1000)	Cell Signaling
Bcl-2	Mouse	SC: 7382	WB (1:1000)	Santa Cruz
Caspase-3	=	SC-7272	WB (1:1000), IF (1:100)	=
β-Actin	Mouse	SC-47778	WB (1:1000)	=

## Data Availability

All of the data is contained within the article.

## Abbreviations

AMPK	5’AMP-activated protein kinase
Bax	Bcl-2-associated X protein
Bcl-2	B-cell lymphoma 2
CD64	Fc γ receptor I
COX-2	Cyclooxygenase-2
DAPI	4′,6-diamidino-2-phenylindole
DG	Dentate gyrus
ES	17β-estradiol
GFAP	Glial fibrillary acidic protein
GST	Glutathione S-transferase
GR	Glutathione reductase
Iba-1	Ionized calcium-binding adaptor molecule 1
IHC	Immunohistochemistry
iNOS	Inducible nitric oxide synthase
Nrf2	Nuclear factor erythroid 2-related factor 2
ROS	Reactive oxygen species
SNAP-23	Synaptosomal-associated protein 23
SWI	Stab wound injury
SYP	Synaptophysin
TGF-β	Transforming growth factor beta
